# Evaluation of coronavirus-2019-related arterial thrombosis in noncontrast spectral computed tomography with electron density imaging

**DOI:** 10.1016/j.radcr.2022.09.085

**Published:** 2022-10-27

**Authors:** Junji Mochizuki, Takeshi Nakaura, Hiroaki Matsumi, Yoshiki Hata

**Affiliations:** a Department of Radiology, Minamino Cardiovascular Hospital, 1-25-1 Hyoue, Hachioji, Tokyo 192-0918, Japan; b Department of Diagnostic Radiology, Graduate School of Medical Sciences, Kumamoto University, Kumamoto, Japan; c Department of Cardiology, Minamino Cardiovascular Hospital, 1-25-1 Hyoue, Hachioji, Tokyo 192-0918, Japan

**Keywords:** COVID-19, Thrombosis, Computed tomography, Electrons, Contrast media

## Abstract

Thrombosis can be associated with coronavirus disease 2019 infection. Computed tomography is essential for the diagnosis of pneumonia in these patients and conventionally contrast agents are required for the assessment of thrombus. In this study, we report a patient with coronavirus disease 2019 who was diagnosed with thrombosis using spectral noncontrast computed tomography with electron density imaging. The patient was a 76-year-old man who presented with a 2-day history of lower-leg pain. Tachycardia and atrial fibrillation were identified, with elevated D-dimer, N-terminal pro-B-type natriuretic peptide, creatine kinase, and C-reactive protein levels. Polymerase chain reaction testing for severe acute respiratory syndrome coronavirus-2 was positive. Conventional computed tomography showed pulmonary changes consistent with coronavirus disease 2019 and no changes in the aorta, but spectral computed tomography with electron density imaging of noncontrast computed tomography showed a thrombus in the right external iliac artery. Spectral computed tomography with electron density imaging provides more data compared with conventional computed tomography and has the potential to depict thrombus without the use of contrast media.

## Introduction

The coronavirus disease-2019 (COVID-19) pandemic continues to threaten populations worldwide, and computed tomography (CT) is essential for the assessment of pneumonia. The typical CT findings associated with COVID-19 are usually peripheral and comprise ground-glass opacities, infiltration, and possibly septal thickening, which appear as a “crazy-paving” pattern [1]. Patients with COVID-19 are reported to be more susceptible to thrombotic disease [2]; however, assessment of thrombosis by CT requires the use of contrast media.

Spectral CT with electron density imaging (EDI) is a form of CT imaging that involves dual-layered detectors to obtain 2 sets of energy data [3]. As a result, spectral CT can obtain ED values that cannot be obtained with conventional CT [3], and the method does not require the use of contrast agents. Contrast agents are associated with adverse events in some patients [4,5], and acute kidney injury has been reported in patients with COVID-19 [6].Therefore, the use of contrast agents is a concern in patients with COVID-19. We report a case of a patient with COVID-19 in whom an arterial thrombus was identified using EDI from spectral CT without the use of contrast media.

## Case report

**Fig. 1:**
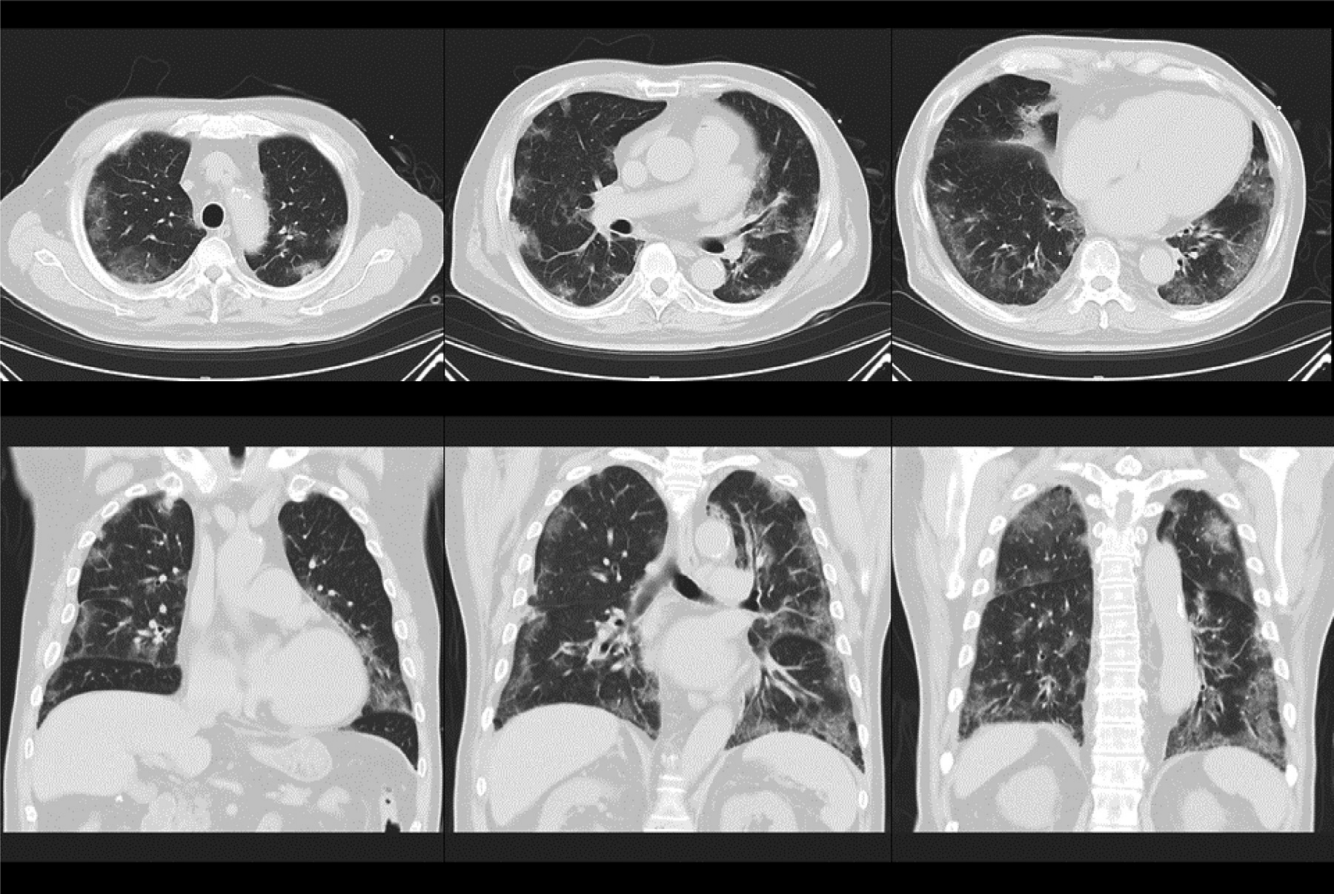
Chest computed tomography (CT) showing bilateral consolidating and infiltrative, peripheral ground-glass opacities, characteristic of coronavirus disease 2019 (COVID-19).

**Fig. 2:**
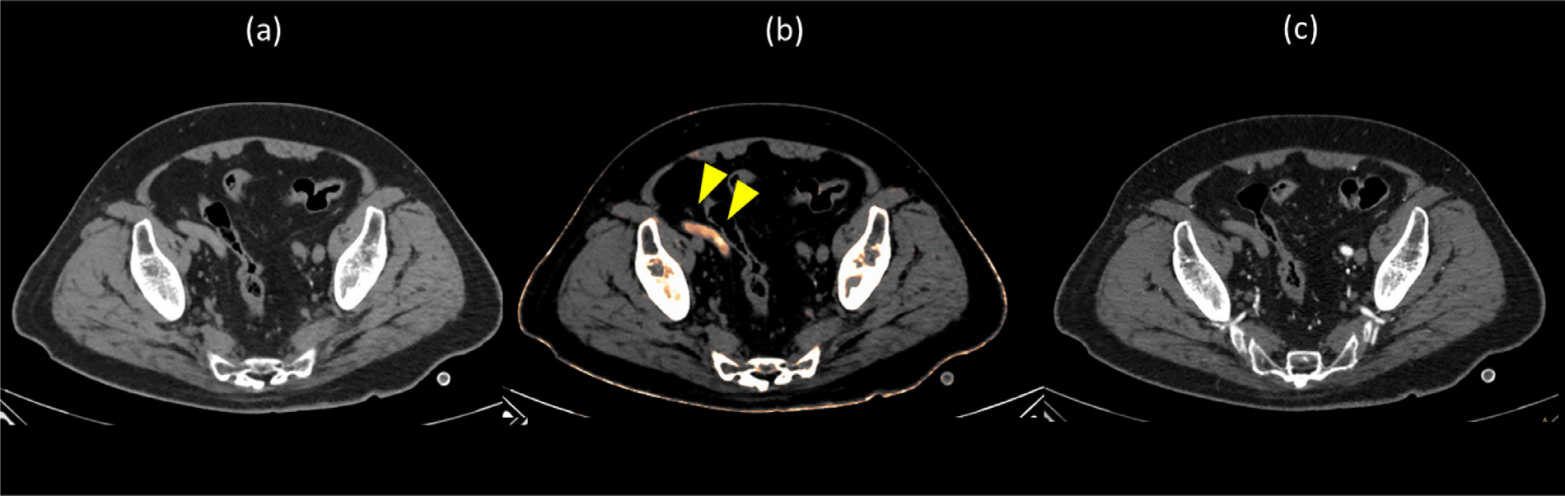
(a) Conventional CT images did not identify areas of high intensity within the arteries in the imaged area; however, electron density imaging clearly identified areas of high intensity (b). The electron density of the right external iliac artery, which showed a high intensity area (yellow arrowheads), was 106.2% of the electron density of water (EDW), while the electron density of the normal left femoral artery was 104.9% of the EDW. Spectral CT number in the right external iliac artery and left external iliac artery were 66.3 HU and 54.2 HU, respectively. Contrast CT showing no contrast effect in the areas of high intensity seen on electron density images (c).

A 76-year-old man presented to the emergency department with a 2-day history of pain in his lower limbs. He had no symptoms related to COVID-19 on presentation. At admission, his heart rate was 170 bpm and he had atrial fibrillation. Blood tests showed a high D-dimer level (66.5 μg/mL, normal 0.00-0.49), suggesting a thrombus, and high N-terminal pro-B-type natriuretic peptide (8720 pg/mL, normal ≤125), creatine kinase (5433 U/L, normal 59-248) and C-reactive protein (13.79 mg/dL, normal 0.00-0.30). A polymerase chain reaction test was performed, and the patient was diagnosed as COVID-19-positive. Transthoracic echocardiography revealed diffuse wall hypokinesia in the left ventricle with an ejection fraction of 30%, and a thrombus in the apex of the left ventricle. Suspecting acute arterial occlusion, spectral CT of the aorta was performed from the thoracic region to the lower branches, with both noncontrast and contrast imaging. Chest CT showed multiple peripheral ground-glass lung opacities with bilateral infiltration, characteristic of COVID-19 pneumonia [1]. (Fig. 1). There were no abnormal findings within the aorta or its branches on conventional CT images (Fig. 2a). Spectral CT with EDI showed hyperintensity in the right external iliac artery, which differed from the intensities in the surrounding arterial and venous vasculature. Treatment for COVID-19 was initiated with a steroid infusion. Following this treatment, amputation of the right lower limb was considered because of the thrombosis. However, the patient had reduced cardiac function and severe obstructive lung impairment. Therefore, the risk of general anesthesia was high, and surgery was not possible. We discussed our findings with the patient, and the implications, and palliative care was elected. Fentanyl citrate therapy was then initiated at a dose of 0.5 mg/kg. The dose was subsequently increased to 1 mg/kg, which provided good pain control; the patient died 16 days later due to complications associated with COVID-19. Biopsy and autopsy were not performed.

## Discussion

**Fig. 3:**
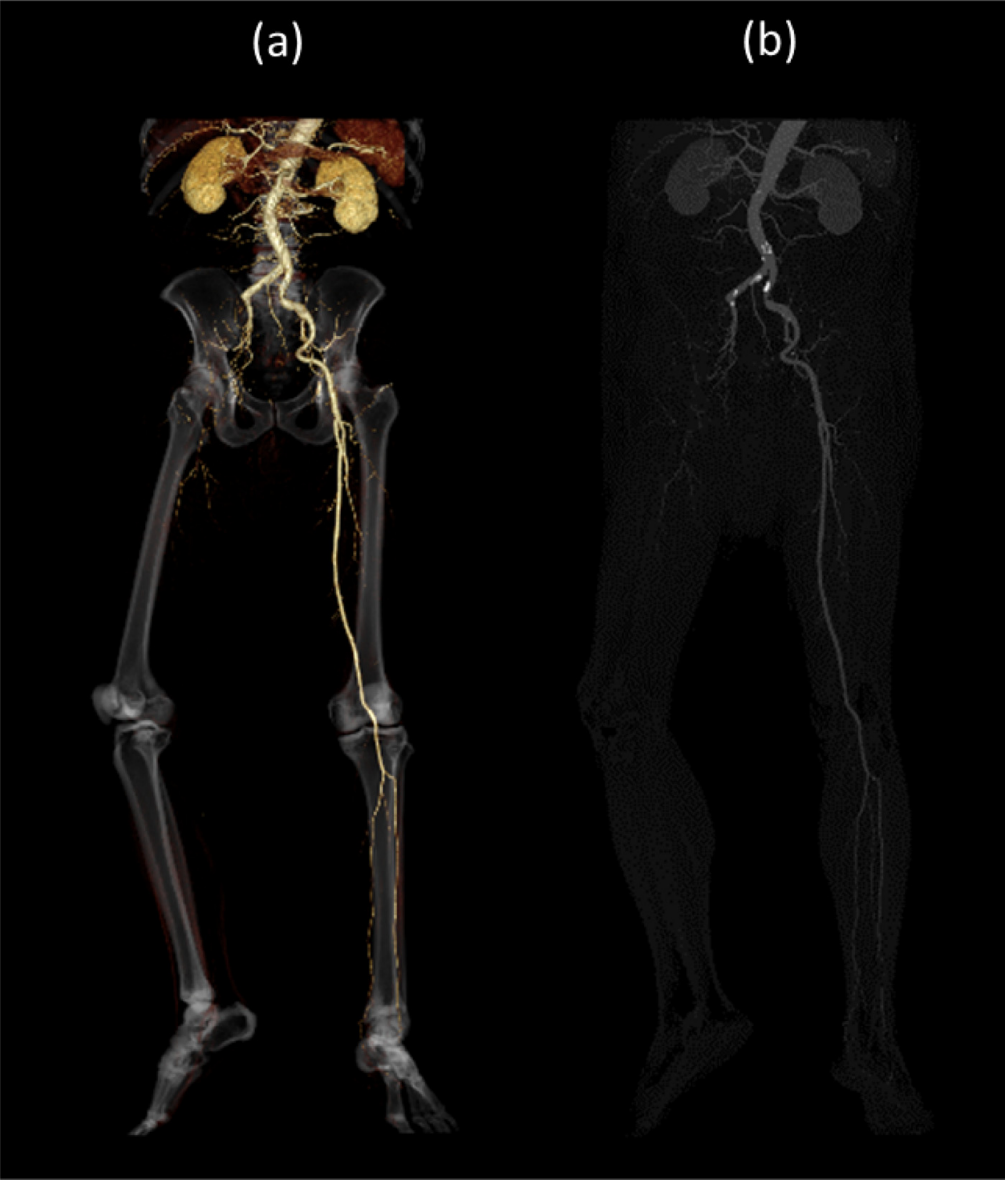
Three-dimensional CT angiography (a, b) showing an arterial occlusion extending from the common iliac artery to the right external iliac artery.

EDI is a spectral imaging technique that measures the electron density index [7]. EDI provides images with different characteristics to those of conventional CT images, and the method has in patients with COVID-19 pneumonia, EDI can improve visualization of parenchymal lung lesions as compared with conventional CT [8]. In a previously reported case, CT EDI was used to rule out pneumonia in a patient in which conventional CT did not reveal hyperintense regions in the ventricles that were subsequently revealed using CT EDI [9]. In the current case, EDI showed hyperintensity in the right external iliac artery that differed from the intensities in the surrounding arterial and venous vasculature (Fig. 2b). Contrast-enhanced CT showed no contrast effect in the same area (Fig. 2c), and an occlusion in the right external iliac artery was identified and diagnosed as an acute arterial occlusion (Fig. 3a and b). In this case, the thrombus could be visualized with contrast CT but not with noncontrast CT. The thrombus could also be visualized using spectral CT with EDI, which does not involve the use of contrast. Because of concerns regarding adverse events associated with contrast agents [4,5], spectral CT with EDI can be very useful. Conventional CT provides CT number only. However, spectral CT provides a variety of images in addition to the conventional CT number, which is one of the reasons we used EDI in this case. Additionally, evaluation of the electron density in arteries may allow identification of thrombi. In patients with COVID-19 and a suspicion of thrombosis, clinicians should be aware of the option to use spectral CT with EDI for thrombosis diagnosis to avoid contrast agents, which may increase the risk of kidney injury.

In conclusion, thrombosis due to COVID-19 can be fatal and should be diagnosed early. Spectral CT is useful in the evaluation of pneumonia, but noncontrast CT with EDI may help in the early diagnosis of thrombosis.

## Patient consent

The patient provided permission to publish the details of his case and for the future use and publication of his images, at the time the images were obtained.
